# Acid whey valorization: novel approaches for probiotic and functional products development

**DOI:** 10.3389/fnut.2025.1630925

**Published:** 2025-09-19

**Authors:** B. Mutaliyeva, E. Turkeyeva, G. Madybekova, L. Živković, VSSL Prasad Talluri, A. Issayeva, M. Vinceković

**Affiliations:** ^1^Department of Biotechnology, M. Auezov South-Kazakhstan University, Shymkent, Kazakhstan; ^2^International University of Tourism and Hospitality, Turkestan, Kazakhstan; ^3^Department of Chemistry, O. Zhanibekov South-Kazakhstan Pedagogical University, Shymkent, Kazakhstan; ^4^Department of Chemistry, Division of Agroecology, Faculty of Agriculture, University of Zagreb, Zagreb, Croatia; ^5^Industry Development Lab, National University of Singapore, Singapore, Singapore; ^6^“One Belt, One Road” Petroleum Engineering Institute, Kazakh-British Technical University, Almaty, Kazakhstan

**Keywords:** acid whey, strategies for acid whey valorization, functional products, fermented beverages, microencapsulation, probiotic viability, acid whey fermentation

## Abstract

Acid whey, a byproduct of dairy processing, particularly from Greek yogurt and cottage cheese production, presents significant environmental and economic challenges due to its high organic load and disposal restrictions. However, the unique composition of acid whey, containing proteins, lactose, minerals, and bioactive compounds, presents promising opportunities for the development of functional, value-added products. This review explores innovative approaches to acid whey valorization, emphasizing biotechnological methods, fermentation techniques, and advanced membrane filtration processes. Comparative analysis of sweet and acid whey compositions underscores specific challenges and advantages in acid whey utilization, various valorization strategies, such as membrane filtration and ultrafiltration, osmosis, enzymatic, and microbial processing, highlighting their effectiveness in ingredient recovery and product development. It also identifies suitable microorganisms capable of efficiently metabolizing acid whey and enhancing the nutritional and functional profiles of derived products. Special attention is given to fermented beverages and other functional products developed from acid whey, incorporating novel strategies to optimize fermentation processes. Moreover, this review details recent advancements in probiotic microencapsulation technologies, demonstrating their effectiveness in maintaining and enhancing probiotic viability in acid whey-based functional beverages. The success of these functional products significantly depends on selecting appropriate probiotic strains, encapsulation materials, and innovative microencapsulation methods. Finally, the article addresses current limitations and outlines future research perspectives, highlighting the potential applications of acid whey-derived products in food, beverages, animal feed, and bioenergy sectors.

## 1 Acid whey utilization challenges

Whey is a residue after cheese production and rich in carbon and nitrogen, that contains high concentration of lactose, proteins and minerals (1–5). Depending on the type of milk coagulation, there are 2 types of whey: rennet whey, which remains after enzymatic milk coagulation, and acid whey, a byproduct of milk coagulation by acidification (6).

According to Solieri et al. (2), there are 2 main ways to use whey:

1. Filtration to obtain useful whey components such as whey permeate, whey protein isolate, whey protein concentrate (WPC), whey powder, or lactose.
2. Biotechnological treatment uses enzymes and microorganisms to convert lactose and whey proteins into useful biochemical.

Also, in addition to biotechnological methods for processing acid and sweet whey due to the high lactose content; chemical methods are also available. For example, Brönsted acid catalysts (hydrochloric acid, nitric acid, sulfuric acid, and phosphoric acid) were used in the acid catalyst experiment (7).

The biotechnological approach to utilize sweet and acid whey is preferable due to cost-effectiveness, and research on whey processing using microorganisms remains easier and more beneficial.

But while sweet whey has commercial uses in products such as nutritional supplements, a major proportion of acid whey from the dairy industry is discarded as effluent, constituting a form of pollution (8). Kazakhstan also has many small dairy industries where acid whey is a co-product after production; however, because of its liquid consistency, it is less suitable for processing, making its utilization more challenging compared to rennet whey.

In the book of Rocha-Mendoza et al. (9) acid whey is described as a waste produced during the production of cottage cheese, and authors Rocha-Mendoza et al. (9) described acid whey after Greek yogurt, and in both productions it has less proteins, contains high salt and acid concentration in comparison with sweet whey, and this cause the problem with further utilization of acidic whey.

There are many types of research devoted to the utilization of acid whey as shown in review of Rocha-Mendoza et al. (9), that concentrated on the industrial trends, applications and health benefits of acid whey products.

For utilization of acid whey usually are used biotechnological way with the cultivation of various cultures of microorganisms and their combinations. For example authors described commercial microorganisms combinations such as *Streptococcus thermophilus*, *Bifidobacterium* spp., *Lactobacillus acidophilus*, *Bifidobacterium lactis*, *Lactobacillus casei*, *Lactobacillus rhamnosus*, *Lactobacillus delbrueckii* subsp. *bulgaricus*, and *Lactococcus lactis* (commercial); 2 combination of microorganisms of home using *Lactobacillus delbrueckii subsp. bulgaricus* and *Streptococcus thermophilus, and Bifidobacterium* spp., *Lactobacillus acidophilus*, *Lactobacillus delbrueckii* subsp. *bulgaricus*, *Lactobacillus paracasei*, and *Streptococcus thermophilus.* At the same time, acid whey, regardless of which combinations of microorganism cultures were used, has approximately the same content of fats, proteins, lactose and medium pH, which indicates metabolic products obtained during fermentation (10). Acid whey *can be c*onverted into functional products, but due to its too-low pH and lactose content, products based on it are mostly liquid. In addition, acid whey’s acidic pH optimum and severe product inhibition limits its application for lactose hydrolysis in milk (11).

Therefore, despite of rich content, the acid whey is a by-product that remains largely underutilized and represents an environmental problem by contributing to water and soil pollution (1, 12). Although numerous works discuss acid whey utilization, its processing remains a significant challenge.

What are the problems with acid whey utilization? Firstly, manufacturers consider acid whey as waste and send it to wastewater, which becomes too expensive to treat due to its high Biochemical Oxygen Demand (BOD) (13).

Secondly, acid whey is high in lactose and low in protein.

Thirdly: Due to the high content of lactic acid, it is difficult to dry acid whey or extract lactose from it (14, 15). But it should be noted that despite the difficulties in processing acid whey, there are advantages in its use in comparison with sweet whey. Although acid whey contains a lower protein content than sweet whey, the presence of approximately double the content of calcium as compared to sweet whey increases the value of acid whey as a source of nutraceuticals (10).

Therefore, there is a trend in utilizing acid whey to produce the useful products and high-value components.

## 2 Valorization of acid whey: challenges and approaches

**Figure 1:**
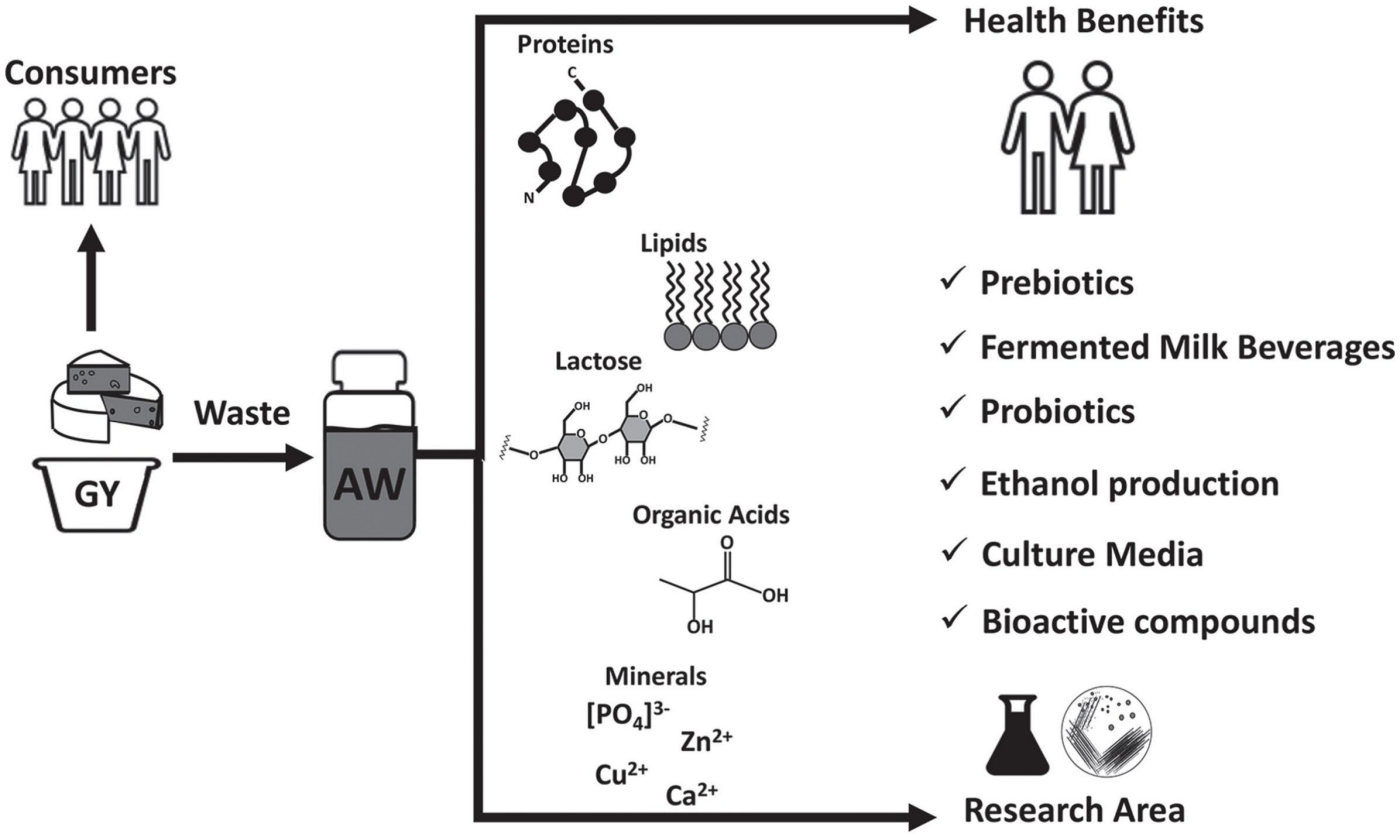
Outline of acid whey uses and its valorization. GY, Greek yogurt; AW, Acid whey. Reproduced from (9). licensed under CC BY-NC-ND 4.0.

Acid whey, which is the byproduct of acid coagulation in cheese production, has traditionally been considered a waste product with limited value. However, recent research has shown that acid whey, a rich source of nutrients, including carbohydrates, proteins, isoflavones, and micronutrients, can be utilized in a variety of high-end products, ranging from beverages to cosmetics (16–19). There is biotechnological approach for utilization acidic whey, and biotransformation of whey can be achieved by biotechnological tools, including microbial fermentation and enzymatic treatment (16) (Figure 1).

For developing value-added uses of dairy co-products such as acid whey waste it is very important to know its content (20). The main components of AW (Greek and Cottage) were 2.1–3.5% of lactose, crude protein content ranged from 1.65, 1.71 to 5.05 mg/g, and low pH ranged from 4.21 to 4.51. Acid whey was characterized also by high chemical oxygen demand varying from 31.900 to 62.400 mg/L; and biochemical oxygen demand ranged from 32.700 to 50.500 mg/L, and highest mineral content (20). This means that low pH, and high levels of lactose – are the most abundant problem for utilizing of acid whey, but at the same time it is possible to use it for the valorization of value-added components.

In addition, acid whey composition has some differences with sweet whey composition, and consists mostly of water, with lactose, high acidity and mineral content, which influence the strategies for their processing (20) (Table 1).

**Table 1:** Comparison of sweet and acid whey composition.

Composition	Acid whey	Sweet whey	Reference
Total solid, % wb	4.27–5.92	60.64 ± 1.15 g/L	(56, 119)
Minerals g/L	3.49–5.04	1.2	(22, 119)
Ca^2+^, wt, %	1.98 wt, % 1.91 ± 0.01b g/L	0.69 wt, % 0.29 ± 0.00 g/L	(22, 119)
Phosphate ions	0.8–0.92 g/L	0.34–0.4 g/L	(22, 119)
Total Proteins % wb	0.16–0.48 7.5 + 0.2 wt%	5.52 ± 0.47 g/L 9.2 + 0.3 wt%	(20, 22, 119)
Lactose % wt	67% Wt 2.1–3.5	72–73% wt 49.44 ± 0.55 g/L	(19, 20, 22)
Lactic acid	7.19–13.07 g/L 14.4 + 1.5 wt%	1.2 + 0.1 wt%	(22, 119)
pH	4.21–4.7	5.9–6.4	(19, 20, 22, 119)
COD mg/L	31.9–62.400	50–102 g/L	(19, 20)
BOD mg/L	32.7–50.5	35–60 g/L 35.000–55.000 mg l^−1^	(19, 20, 22)
Ash % wt	0.6–0.73% wt	0.5% wt 4.41 g/L	(22, 119)

Because of its rich content in protein and excellent amino acid profile production of high-quality protein powders is one of the potential uses of acid whey (9). Therefore protein powders made from acid whey could be marketed as a premium product, given their high nutritional value and eco-friendly sourcing (21).

At the same time the whey powder production from Acid whey is more problematic compared with Sweet whey because AW has a more acidic environment due to the high content of organic acids, and lower content of lactose and proteins (22).

However, as the authors note (22), the higher content of minerals and organic acids contributes to a higher rate of nanofiltration followed by electrodialysis, so reduction of ions from the acid whey is observed to be more intensive than from sweet whey.

Whey concentrate produced as a product of acid whey utilization is an ingredient of nonfat yogurt (12). There is the potential for recovery of whey protein from cottage cheese acid whey for use in yogurt. To overcome the low pH of acid whey food-grade ammonium hydroxide can be used to reach pH 6.4, then the whey is heated to 50°C and concentrated using ultrafiltration and diafiltration, for isolation of acid whey protein concentrate, allowed to use this protein in the producing of yogurt with gel-like texture.

Some works also considered the potential use of acid whey for the production of dairy products such as kefir and yogurt (15).

Another problem of acid whey is the high content of lactose that can be utilized by probiotic bacteria to produce lactic acid, which helps to lower the pH of the product and give it a tangy flavor (6). Fermented dairy products made with acid whey could be marketed as a premium, sustainable alternative to traditional products. Acid whey can also be used in the production of alcoholic beverages, such as beer and spirits (23). The lactose in acid whey can be fermented by yeast to produce alcohol, while the whey protein can contribute to the texture and mouthfeel of the finished product.

Finally, acid whey can be utilized in the production of cosmetics and personal care products (24). The proteins and other components in acid whey can have moisturizing and anti-aging properties, making it a potentially valuable ingredient in skincare and haircare products.

Some biotechnological approaches are devoted to using acid whey as substrate for converting lactose into galactooligosaccharides (GOS) with prebiotic activity using commercial *β*-galactosidases or microorganisms with high β-galactosidase activity, and products produced during valorization can be used for people with lactose intolerance (25–28).

Some works described that treatment of whey leads to the release of nutraceuticals such as bioactive peptides, prebiotics, exopolysaccharides, organic acids, bacteriocins, isoflavone aglycones, and to the production of industrially important enzymes including *β*-galactosidase, protease, and amylase, and development of novel functional foods with health beneficial effects (18, 19).

The mechanism of producing the *β*-galactosidase enzyme from lactic acid bacteria using AW as a nutrient medium substrate is considered by Kolev et al. (29). They showed the potential of using acid whey for many purposes, such as the production of β-galactosidase to obtain lactose-free products, to produce prebiotic galactooligosaccharides, and to reduce the BOD of acid whey before its disposal. In addition, it has been shown that acid whey itself may be a more hospitable environment for lactic acid bacteria (LAB) than both sweet whey and de Man, Rogosa, and Sharpe medium (MRS), because it has a high content of vitamins, minerals, and most importantly, lactose. It is proposed to use a culture of lactobacilli with yeast extract as a source of proteins or nitrogen (29).

For people who are lactose intolerant, it is important to eat lactose-free foods. Because acid whey is rich in carbohydrates, the selection of microorganisms capable of utilizing it as a substrate for the bioconversion of lactose into galactooligosaccharides is an important direction for the whey valorization and functional products development.

Besides, biotransformation of whey carbohydrates into prebiotics by recombinant enzymes has proven effective for the valorization of whey (18). Efficient conversion of whey lactose to GOS was obtained using recombinant *Streptococcus thermophilus β*-galactosidase expressed in *Lactobacillus plantarum* (30). Moreover, commercially available enzymes such as lactozyme 2,600 L, obtained from *Kluyveromyces lactis* have demonstrated high lactose conversion rates with observed GOS yields of up to 25% (19).

Some authors considered attempts to convert AW to biogas, ethanol, or lactose food products and they concluded that these attempts have all been hindered by the presence of lactic acid and high capital investment requirements, which restrict AW conversion in small-scale dairies. Using whey lactose and lactate for polyhydroxyalkanoates (PHAs) production is an opportunity that would not only provide low-cost renewable feedstock without competing with edible products but also simultaneously solve an environmental problem (31). Some *Escherichia coli* strains are acid-tolerant (28). But using AW as substrate has challenges (31): one challenge is the need for pretreatment of lactose in AW to obtain sugars that are more readily fermented and increase the carbon-to-nitrogen ratio (C/N); the second major challenge specific to the AW utilization is the low pH, due to the presence of lactic acid (LA). Another challenge is the need to maintain a fermentation pH above 7 to sustain PHA synthase enzymes. Knowledge gaps limiting the high-yield conversion of AW to PHAs are: pH control, eliminating pretreatment of lactose, microorganisms that will consume both lactose and LA, increasing the C/N ratio, and a sustainable source for process additives like salts and minerals (Table 2).

**Table 2:** Strategies to valorize of acid whey.

Source	Research	Product	Method	Strain/enzyme	Key findings
(10)	Acid whey metabolomics	Metabolic profile	Analytical	-	Acid whey has a rich metabolic composition
(120)	Formulating ranch dressing	Formulating ranch dressing by replacing buttermilk with Yogurt Acid Whey (YAW). No lactic acid was added (lactic acid is naturally present in YAW)	Food technology	-	Up to 60% of buttermilk can be replaced by acid whey
(53)	GOS synthesis	GOS	Microbial/enzymatic	*Cryptococcus laurentii*, *Aspergillus oryzae*	Acid whey as substrate for GOS synthesis
(121)	prebiotic GOS	Prebiotic galacto-oligosaccharides production from acid whey	Enzymatic	β-galactosidases (from *Kluyvero-myces lactis* and *Aspergillus oryzae*) and one novel, in-house produced (from *Thermothielavioi-des terrestris*),	Efficient, cost-effective production of valuable prebiotics from acid whey. The maximum GOS yield was 25.7% when using concentrated acid whey with 20% lactose content and the enzyme from *T. terrestris.*
(31)	Production of Polyhydroxybu-tyrate (PHB) by use of Recombinant *Escherichia coli* LSBJ	Polyhydroxybuty-rate (bioplastic)	Microbio-logical	Recombinant *Escherichia coli* LSBJ	Bioconversion of acid whey (AW) to PHB
(29)	β-galactosidase	Biocatalysis	Enzymatic	LAB, *Bifidobacterium* spp.	LAB strains assessed for enzyme activity in acid whey
(6)	Fermented beverages based on processing of acid whey.	Fermented probiotic beverages The production process included combining pasteurized acid whey with UHT milk, unsweetenedcondensed milk or skim milk powder-introduced milk to enrich casein content and obtain a product with characteristics similar to that of fermented milk drinks.	Microbiolo-gical	*Lactobacillus* *acidophilus* LA-5 or *Bifidobacterium animalis ssp. lactis* BB-12	Acid whey is a suitable base for functional beverages. *L. acidophilus* provides more acidity of beverages in comparison with *B. animalis.*
(12)	Functional ingredients for yogurt	Acid whey protein concentrate after neutralization by food-grade ammonium hydroxide, and then ultrafiltration and diafiltration.	Technolo-gical	—	Whey protein concentrate is used for yogurt making, stable sensory and physicoche-mical properties
(25)	GOS	GOS from acid whey	Enzymatic	β-galactosidase from *Aspergillus oryzae* and *Kluyveromyces lactis* *Trichoderma terrestris*	Enzymes from *Aspergillus oryzae* and *Kluyveromyces lactis,* *Trichoderma terrestris* have thermostable and acid pH-stable β-galactosidase.
(13)	Bioenergy	Medium-chain carboxylic acid	Microbio-logical	Microbial composition	Bioconversion of acid whey
(64)	Probiotic beverage	Whey and pineapple juice drink	Microbio-logical	*Lactobacillus acidophilus*, *Bifidobacterium bifidum*	Probiotic beverage with good sensory properties
(4)	Functional beverage	Fermented beverage	Microbio-logical	LAB	Probiotic potential and antioxidant properties
(29)	β-galactosidase activity	Hydrolysis of acid whey	Microbiolo-gical/Enzy-matic	LAB strains	Acid whey valorization using LAB enzyme potential
(3)	Fermented probiotic beverage	Fermented probiotic beverage	Microbio-logical	LAB and yeast	Enhanced antimicrobial and antioxidant properties
(23)	Functional beverage	Beverage based on acid whey	Microbio-logical	*Brettanomyces claussenii*	Lactose conversion
(9)	Review of acid whey benefits	Multiple functional foods	Review	-	Acid whey has health potential and industrial uses
(2)	Bioactive peptides	Functional peptides	Microbio-logical	*Streptococcus thermophilus*	Biopeptides
(27)	β-galactosidase optimization	Milk and whey processing	Enzymatic	β-galactosidase (acid- and cold-tolerant)	Enzyme suitable for acidic whey conditions
(49)	Whey spirit production	Fermented acid whey spirits	Microbio-logical/ Distillation	Fermenting yeast strains	Characterized volatile compounds during distillation stages
(22)	Membrane processing	Treated acid whey	Technolo-gical	—	Electrodialysis and nanofiltration alter whey composition
(119)	Mineral removal from acid whey	Calcium and lactate extraction	Technolo-gical	—	Electrodialysis effective in adjusting mineral content
(7)	Chemical conversion	5-HMF and levulinic acid	Chemical / Thermoche-mical	—	Optimized synthesis from acid whey and lactose
(47)	Exopolysaccharide production in whey	Iron-complexing EPS	Microbio-logical	Lactic acid bacteria	LAB produce EPS in acid whey with iron-binding properties
(122)	Whey fermentation promoters	Genetic regulation insights	Microbio-logical	—	New regulatory promoters discovered in acid whey fermentation
(16)	Value of acid whey stream	Functional ingredients	Review	—	Acid whey is becoming more valuable than the main dairy product
(19)	Microbial and enzymatic valorization	Nutraceuticals: bioactive peptides, prebiotics, exopolysaccharides, organic acids, bacteriocins, isoflavone aglycones; and industrially important enzymes: *β*-galactosidase, protease, and amylase. Whey enriched with bioactive compounds can be utilized for the functional and nutritional enhancement of foods and the development of novel functional foods with health beneficial effects.	Microbial and enzymatic	LAB, enzymes	Acid whey enriched with bioactive compounds can be utilized for the functional and nutritional enhancement of foods and the development of novel functional foods with health beneficial effects.
(26)	GOS synthesis from whey	Galactooligosaccharides	Enzymatic	lactases from *Aspergillus oryzae* and *Kluyveromyces lactis*	Both acid and sweet whey are suitable for GOS production
(24)	Cosmetic biotechnology	Innovative cosmetic product for hair based on lactoserum	Technolo-gical	—	Whey based concentrates in cosmetics
(20)	Composition analysis	Acid whey from Greek yogurt (GAW), acid whey from cottage cheese (CAW), and milk permeate (MP).	Analytical	-	Composition data of coproduct streams: acid whey from Greek yogurt (GAW), acid whey from cottage cheese (CAW), and milk permeate (MP).
(34)	Membrane technology applications	Dairy coproducts	Techno-logical	—	Membrane processes used for treating acid whey
(32)	Historical and future outlook	Acid whey	Review	—	Trends and possibilities for whey valorization
(11)	Engineering the optimum pH galactosidase	Glycoside hydrolase	Engineering	*Aspergillus oryzae*	Y138F and Y364F mutants exhibited better hydrolytic ability than lacA in milk lactose hydrolysis.
(28)	GOS	GOS	Enzymatic/microbio-logical	*Aspergillus oryzae* galactosidase, *Kluyveromyces marxianus* and *Saccharomyces cerevisiae* cells	Reacted medium without nutrient supplementation (raw GOS) was fermented *with Kluyveromyces marxianus* cells obtaining GOS of 95% purity containing mostly tri- and tetrasaccharides with total recovery of GOS after 24 h.

## 3 Valorization strategies using membrane filtration and ultrafiltration

Advanced filtration techniques can separate valuable components such as proteins and minerals from acid whey. Ultrafiltration and reverse osmosis enhance protein recovery while reducing waste volume.

Advanced filtration techniques, such as ultrafiltration (UF), nanofiltration (NF), and reverse osmosis (RO), play a crucial role in separating valuable components from acid whey, reducing waste, and improving its functional applications. These membrane-based technologies enable the selective recovery of proteins, minerals, and lactose, transforming acid whey from an environmental burden into a resource for functional food and ingredient development.

### 3.1 Ultrafiltration for protein recovery

Ultrafiltration is commonly employed to concentrate and isolate proteins from acid whey. The membrane pore size (typically 1–100 nm) allows the retention of high-molecular-weight proteins, such as *α*-lactalbumin and *β*-lactoglobulin, while permitting the passage of smaller molecules like lactose, minerals, and organic acids (32). The protein fraction obtained through UF has high nutritional and functional value and can be used in sports nutrition, infant formula, and functional food applications (33).

### 3.2 Nanofiltration for partial desalination

Nanofiltration is used after UF to remove excess minerals from the acid whey stream while retaining valuable peptides and carbohydrates. The partial removal of minerals improves the sensory properties of acid whey-derived ingredients, making them more suitable for incorporation into food formulations (34, 35).

### 3.3 Reverse osmosis for concentration and waste reduction

Reverse osmosis, with its tighter membrane structure, effectively concentrates acid whey by removing water, reducing transportation and storage costs. This process enhances the sustainability of acid whey valorization by minimizing waste volume and facilitating further processing into powdered whey ingredients (36). The combination of UF, NF, and RO can achieve up to 90% water recovery, making acid whey processing more environmentally sustainable (18, 37).

### 3.4 Emerging membrane technologies

Innovative membrane-based techniques such as electrodialysis (ED) and forward osmosis (FO) are gaining attention for selective separation and demineralization of acid whey, enabling its application in beverages, dairy alternatives, and nutraceuticals (38). These advancements contribute to the circular economy approach in dairy processing.

Advanced filtration techniques provide a sustainable and economically viable solution for acid whey valorization. The integration of UF, NF, and RO, alongside emerging membrane technologies, enhances the functional properties of acid whey by concentrating valuable proteins and reducing waste. Continued research and optimization of membrane processes will further improve the efficiency and commercial feasibility of acid whey-based functional product development.

## 4 Microorganisms for acid whey utilization

Lactic Acid Bacteria (LAB) for Fermentation.

Lactic acid bacteria are naturally present in acid whey and can be employed for fermentation-based valorization. LAB such as *Lactobacillus delbrueckii*, *Lactobacillus plantarum*, and *Streptococcus thermophilus* efficiently utilize lactose and lactic acid from acid whey, converting them into lactic acid which is a precursor for biodegradable plastics and food preservatives (39). Exopolysaccharides (EPS), enhance the texture of dairy products and serve as prebiotics (40). LAB-driven fermentation can reduce acid whey’s chemical oxygen demand (COD), mitigating its environmental impact while producing commercially valuable compounds.

### 4.1 Yeasts for ethanol and biopolymer production

Certain yeast strains can metabolize lactose and organic acids in acid whey, leading to bioethanol production, an important biofuel alternative. *Kluyveromyces marxianus* is widely studied due to its ability to ferment lactose directly into ethanol (41). *Saccharomyces cerevisiae* can be used with pre-hydrolyzed whey to produce ethanol efficiently (42). Yeasts can also synthesize biopolymers such as polyhydroxyalkanoates (PHA), which serve as biodegradable plastics. *Metabolic engineering* approaches are enhancing microbial conversion efficiencies for sustainable bioprocessing of acid whey (43).

### 4.2 Filamentous fungi for protein and enzyme production

Filamentous fungi such as *Aspergillus oryzae* and *Rhizopus oligosporus* can utilize acid whey components to produce Single-cell proteins (SCPs) for animal feed and food applications (44). Enzymes such as proteases, lipases, and lactases, which are used in dairy and food processing industries (45). These fungi improve the nutritional profile of acid whey, making it a valuable ingredient in biorefinery approaches to waste management.

### 4.3 Microbial consortia for biogas and biohydrogen production

Mixed microbial communities have been successfully employed to convert acid whey into biogas (methane) and biohydrogen: Anaerobic digestion of acid whey by methanogenic consortia yields biomethane, a renewable energy source (46). *Clostridium* species can ferment acid whey to produce biofuel with significant energy potential (43). These microbial-driven approaches promote circular economy models by utilizing acid whey for sustainable energy production.

Microorganisms play a pivotal role in acid whey valorization, offering diverse biotechnological applications such as fermentation for functional food ingredients, biofuel production, enzyme synthesis, and biogas generation. Future research should focus on genetic and metabolic engineering of microbial strains to enhance their efficiency in acid whey bioconversion while optimizing industrial-scale fermentation processes.

Several types of microorganisms can utilize acid whey for various purposes, such as producing value-added products or treating waste.

Among microorganisms’ lactic acid bacteria are most used because of their ability to ferment lactose. LAB are commonly used to ferment acid whey into products such as kefir, yogurt, and sour cream. These bacteria convert lactose, a major component of acid whey, into lactic acid, which lowers the pH and gives the product a tangy flavor. LAB can also produce other compounds such as exopolysaccharides, which can enhance the texture and stability of dairy products (47).

*Lactobacillus bulgaricus* and *Streptococcus thermophilus* are two species of lactic acid bacteria commonly used in the production of yogurt and other fermented dairy products. They can utilize lactose in acid whey to produce lactic acid, which contributes to the characteristic tangy flavor of yogurt.

*Propionibacterium freudenreichii* is a species of propionic acid bacteria that can be used to produce Swiss cheese. It can utilize the lactose and other components of acid whey to produce propionic acid and carbon dioxide, which contribute to the characteristic flavor and texture of Swiss cheese.

Yeasts - Some yeast strains can use lactose in acid whey as a carbon source to produce ethanol and other organic compounds (48). This process is called alcoholic fermentation and can be used to produce alcoholic beverages such as beer or distilled spirits.

*Saccharomyces cerevisiae*s commonly used in the production of beer, wine, and other alcoholic beverages. It can ferment the lactose in acid whey to produce ethanol and other organic compounds, which can be used to produce premium alcoholic beverages (49). *Brettanomyces* and *Kluyveromyces* genera as potential species to produce acetic acid and ethanol from acid whey due to high ability lactose metabolize (50). *Aureobasidium pullulans* is yeast like fungus utilizes lactose from acid whey for the production of polysaccharide polymer called pullulan (51).

Methanogenic archaea - These microorganisms can convert the organic matter in acid whey into biogas, which is mainly composed of methane and carbon dioxide (52). This process is called anaerobic digestion and can be used to treat waste and generate energy.

Fungi - Certain filamentous fungal strains can use acid whey as a substrate to produce enzymes such as proteases, amylases, and lipases. These enzymes can have various industrial applications such as in the food, textile, and detergent industries. *Aspergillus oryzae* is a filamentous fungi and *Cryptococcus laurentii* used in the production of functional sugars and related value-added compounds (53). It can utilize lactose and other components of acid whey to produce enzymes such as proteases and amylases, which can be used in various industrial applications such as food processing and detergent manufacturing.

**Figure 2:**
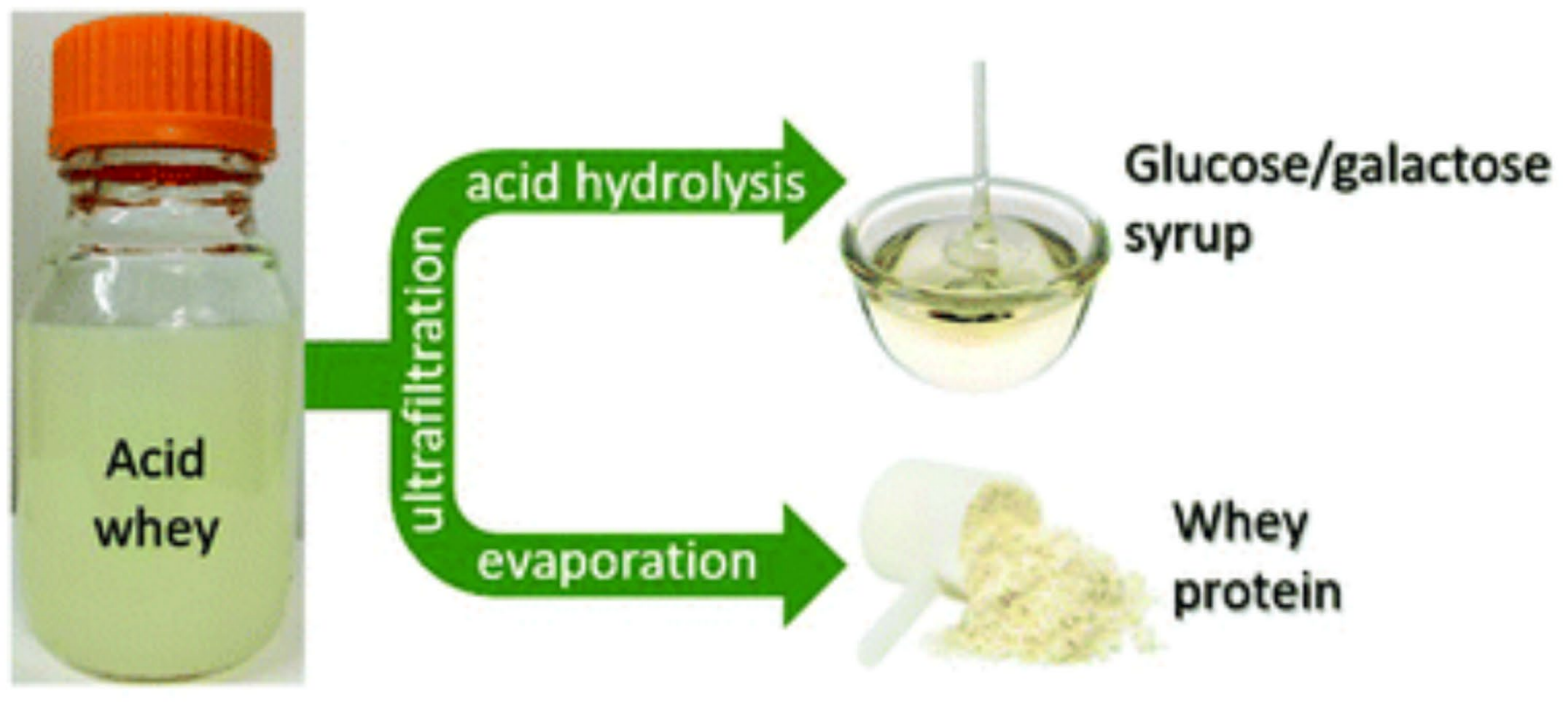
Graphical representation of acid whey utilization pathways. Reproduced from (118), with permission from The Royal Society of Chemistry.

In summary, acid whey can be a valuable substrate for several types of microorganisms, which can be converted it into useful products or treated it as waste. Harnessing the potential of these microorganisms can lead to the development of more sustainable and efficient processes for utilizing acid whey (Figure 2).

Kaur et al. works devoted to utilizing whey with using of microorganisms such as proteolytic LAB strains, yeasts, *Bacillus* spp., fungi, and algae, and treatment of whey by enzymes including *β*-galactosidase, protease, dextransucrase, levansucrase, and *β*-glucosidase that can produce several bioactive compounds with functional properties, and novel functional food products (18).

For example, enzymes from *Aspergillus* oryzae and *Kluyveromyces* lactis have been used to convert lactose using acid whey. Zerva et al. noted that A thermostable *β*-galactosidase (TtbGal1) from *Thermothielavioides terrestris* can be used for the valorization of acid whey, with minimal preparation of the starting material, because this microorganism has a thermostable and acid pH-stable β-galactosidase, and GOS yields reached up 14.8% (25).

Besides LAB, β-galactosidase synthesizing microorganisms such as yeast strains *Kluyveromyces marxianus*, *Kluyveromyces lactis*, fungi *Aspergillus oryzae*, bacteria species *Bacillus circulans* can be used for the production of GOS from whey (11, 19, 26, 28).

Wherry et al. (12) showed that *Aspergillus oryzae is* one of the most important fungal sources of *β*-galactosidase, and lacA produced by this fungi has a pH optimum of 4.5 (54), which is appropriate for lactose hydrolysis of acid whey. The authors noted that β-galactosidase hydrolyzes lactose into its monosaccharide constituents, glucose and galactose, in the small intestine and in the dairy industry, this enzyme is widely used to produce lactose-free milk for people with lactose intolerance.

## 5 Acid whey fermented beverages and functional products -new ways to increase acid whey fermentation

Many papers are devoted to producing beverages based on sweet whey. In the paper authors (21) noted that “One possibility is the generation of whey-based beverages, which can be divided into non-fermented and fermented beverages. With the first, whey is often mixed with fruit juices to obtain a pleasant taste (55). As the authors note (56), various microorganisms were used as starter cultures in a number of studies: typical yogurt cultures (57); probiotic bacteria (58), yeasts (59), and kefir grains (60).

Kaziullayeva et al. (61) proposed using a whey as a substrate for the production of probiotic whey drinks with the use for fermentation of the lactic acid bacteria and yeast resulting in increased antimicrobial, antioxidative, and metabolomics profile of the whey-based beverage. They showed an effective way of whey valorization for industrial stakeholders, because the drink exhibited good antimicrobial activity against five pathogens, and lactic acid bacteria-yeast fermentation improved the antioxidative activity of drink.

Jitpakdee et al. (4) treated whey to produce a functional fermented whey (FW) beverage by using LAB, *Pediococcus pentosaceus* ENM104 and *Lactiplantibacillus plantarum* SPS109. Both strains grew well in MRS medium under aerobic and anaerobic conditions, were susceptible to antibiotics, and showed antibacterial activity via metabolites, i.e., organic acids and produced biofilm. Both LAB in a mixed starter showed great potential to produce functional FW through their joint beneficial activities, and a functional fermented whey (FW) beverage inhibited *Bacillus cereus* TISTR 687, *Listeria monocytogenes* DMST 17303, and *Streptococcus mutans* ATCC 25175 with minimum inhibitory concentration (MIC) from 30 to 40% and biofilm inhibitory concentration (BIC) from 35 to 40%.

Very interesting way to use the fermented whey described by authors of work (61) where their results showed that fermented whey can be used as an environmentally friendly descaling agent.

As an Skryplonek et al. (6) described the properties of whey and whey proteins including antioxidant properties; antimicrobial, immune-stimulating, and anticancer features; reduction of blood pressure and the risk of cardiovascular disease; and satiety regulation, allowed to conclude that whey is a very potential substrate for probiotic fermented beverages production.

Functionality, Formulations, Health Benefits, and Applications of whey-based beverages are described in the work of Chavan (62), in which discussed that whey beverages based on fruit juices, where mixtures of fruit juices and unprocessed or deproteinated whey or UF permeates are the most common types of whey drinks to be manufactured. These beverages are usually used as citrus fruits, mango, passion fruit, pear, apple, strawberry, raspberry, etc., because they can provide covering for the undesirable odor of whey.

Nielsen et al. (36) revealed that the addition in whey of only 1% v/v orange juice or 1% v/v molasses - 4% w/v orange peel reduced the fermentation time to 10 from 70 h is connected with their promotional activity on whey fermentation. Also was observed that a composite biocatalyst consisted of cells entrapped with orange peel in starch gel.

The work found new ways to increase whey fermentation by using orange juice, orange peel and molasses to increase activity.

It has been found that the use of whey in mixtures with low-cost raw materials and the application of a continuous process promote effectively its fermentation by kefir, leading to the potential production of novel alcoholic drinks. The authors reveals new findings concerning the substantial promotional activity of orange juice, peel, and molasses on whey fermentation using single-cell culture of kefir microorganisms, to increase the capacity of whey exploitation and to create a new research concept.

Durpekova et al.(8) described that acid whey can be used as a base for a set of renewable and biodegradable hydrogels mixed with cellulose derivatives and blended with poly (lactic acid) (PLA), and that are designed as eco-friendly biopolymeric material for sustainable agricultural applications. In this study, the novel material for hydrogel production is acid whey, a by-product of the dairy industry arising from the manufacture of cream cheese and Greek yogurt. Because acid whey is usually considered an unused waste, there are many challenges for way how to utilize this unpleasant for ecology by-product. But there are investigations devoted to solving this problems with using of acid whey. In this work, acid whey has been used as a base material of hydrogel to find its effective use and address the associated pollution, whereas contemporary hydrogel production utilizes water instead. Akay and Sert (63) also described that acid whey has high nutrient content, and can potentially be used in fertilization practices because of its positive effect on soil biological properties. Another benefit of acid whey is to correct the pH of alkaline soils through its inherent acidic pH (4.0–5.1).

Rocha-Mendoza et al. (9) concluded that acid whey-based fermented products can be modified by adding flavor ingredients to make whey-supplemented beverages more attractive.

Authors (6) studied the organoleptic and physic-chemical parameters of beverages based on the processing of acid whey. *Lactobacillus acidophilus* provide more acidity of beverages in comparison with *Bifidobacterium animalis.* Beverages based on acid whey and skim milk powder are characterized by higher acetaldehyde content, and beverages made from whey, milk, condensed milk, and *Lactobacillus acidophilus*, had the best sensory properties.

The authors concluded that acid whey was a good medium for probiotic bacteria and grew very well and their concentration was higher than the therapeutic dose.

Islam et al. (64) suggested using LAB as a starter culture to produce probiotic beverages by using whey and pineapple juice, and the proportion 25:75 contributed to the highest sensory score. Because acid whey is more watery, this is problematic to use them for fermented beverage production, but to improve textural charactersitics whey can be combined with fresh milk, condensed milk, or milk powder. As authors noted LAB and *Bifidumbacteruim* can be used for fermentation acid whey (6).

## 6 Ways to increase probiotic viability of functional beverages by use of microencapsulation

Functional beverages are beverages fortified with bioactive substances having health-promoting or disease-preventing properties. Incorporation of bioactive compounds into beverages is a technologically demanding process concerning maintaining their bioactivity and adequate delivery to the organism. The significant bioactive substances are probiotic bacteria which are beneficial to the host only when administered in adequate amounts (65). Effective incorporation of probiotic strains into functional beverages should ensure their stability and survival inside the product as well to pass through the stomach until delivery in the intestine (66). The main problem that occurs during the processing and storage of probiotic bacteria is their reduced viability caused by sensitivity to oxygen, acidity, pH, temperature, and moisture, as well as possible interactions with beverage constituents. Traditional techniques for maintaining the survival of probiotics during processing and storage are the selection of resistant strains, inclusion of protective compounds, oxygen control, adaptation to stress, two-stage fermentation and addition of prebiotic substances (67).

Krunić et al. (5) had the idea that enriching alginate matrix used for probiotic encapsulation with whey as substrate can impact on antioxidant capacity and stability of whey-based beverages. So, the research describes whey that has been used as a substrate for fermentation to create a functional beverage and to solve environmental problems such as water contamination by whey which is a by-product after cheese production, and its utilization can lead to the production of various useful products because of rich and healthy whey content. In addition, this work emphasizes that encapsulation is a solution to the problem of not only preserving the viability of probiotics but also can increase the antioxidant properties of functional beverages.

Encapsulation is an advanced technology and the most effective way of incorporating probiotics into functional food and drink (68). Encapsulation is an entrapment process of a substance (called active agent or component, core material, filling content, internal phase) within an encapsulating material that protects the core material against the surrounding environment. Materials encapsulating the bioactive agent are referred to as matrix, coating material, membrane, or wall material (one wall or more shells arranged in layers of different thicknesses around the core) forming a barrier between the entrapped bioactive and its surroundings. The role of encapsulation of probiotic microorganisms is to ensure survival during processing and storage and protect them from the harmful effects of gastric pH and bile salts to ensure maximum bioavailability to the host (69). Desirable health benefits can only be achieved with a probiotic that has survived passing through the gastrointestinal tract (GIT) in large quantities (10^6^–10^7^ cfu (colony forming unit)/g) (70).

The composition of encapsulating material is the main determinant of microcapsule functional properties and effective use of the bioactive ingredient (71). The selection criteria for the matrix material are the required functionality of the final product, possible limitations of the matrix material, encapsulated concentration, corresponding stability, release kinetics and mechanisms, and economic limitations (72, 73). Hassan et al. (74) summarized the literature available on the applied biomaterials and encapsulation method on probiotics for the development of functional beverage products. Selected material must be biodegradable and food grade, protect probiotics from their surroundings as well must have characteristic physicochemical properties (be inert toward the encapsulated material and other processing ingredients, have good rheological characteristics at high concentration, enable targeted release, etc.). Controlled release from microcapsules allows probiotics safe pass through the highly acidic environment of the stomach and to release in the intestine, which has an alkaline pH.

Various biopolymers such as polysaccharides and proteins, or a mixture of both, are regarded as suitable materials that retain their structure during their pass through the highly acidic environment of the stomach and deliver them in the intestine (75, 76). The selection of the encapsulation technique depends on the required particle average size, the physical and chemical properties of the carrier material, the applications of the encapsulated material, the required release mechanism, and the cost (77). Due sensitivity of probiotics it is also important to choose the mild encapsulation technique. Currently, common probiotic encapsulation techniques are spray drying, extrusion, emulsion, coacervation, lyophilization etc. (78).

Among polysaccharides used for encapsulation (alginate, chitosan, agar, carrageenan, gum arabic, dextrans, xanthan and cellulose (ethyl-cellulose, acetyl-cellulose, methyl-cellulose, carboxymethyl-cellu-lose, nitrocellulose) alginate is commonly used to encapsulate probiotics by various methods such as extrusion, emulsion and spray drying (especially freeze drying) (79–81). Reasons for frequent use are its low price, mild gelling conditions, biocompatibility, GRAS (generally recognized as safe) status, and lack of toxicity. The increasing survival of encapsulated probiotics in alginate particles was confirmed in several studies. Cook et al. (82) gave an overview of the literature available on the alginate particles loaded with the most widely used probiotics. For instance, probiotic bacteria (*Lactobacillus rhamnosus*, *Bifidobacterium longum*, *Lactobacillus salivarius*, *Lactiplantibacillus plantarum*, *Lactobacillus acidophilus*, *Lacticaseibacillus paracasei*, *Bifidobacterium animalis* subsp. *lactis type* Bi-04 *and Bifidobacterium animalis* subsp. *lactis*) type Bi-07 encapsulated in alginate particles revealed much higher survival in fruit juices compared to fruit juices containing free probiotic bacteria (83).

The advantages of alginate as a matrix are its ability to form gels with relatively good mechanical properties, high porosity and biodegradability, easy manipulation as well as good biocompatibility. At very low pH values, the use of the alginate encapsulation matrix alone is limited due to fast degradation and rapid release of encapsulated agents (84). These are the reasons why alginate microparticles are often not sufficient for the encapsulation of bioactive. The functionality of alginate particles and their ability to preserve probiotics bioavailability and assure minimum effective concentration can be adjusted by using alginate-based composites, i.e., by mixing alginate gels with other biopolymers (hydrocolloids, proteins, or starches) in correct proportions (85, 86).

Coating of alginate microspheres with oppositely charged polyelectrolyte, like chitosan, increases microparticle chemical and mechanical stability improving the effectiveness of encapsulation, reducing porosity, limiting the adverse effect of functional food constituents (for example the transition of acids and flavonoids from the fruit juice into microcapsules), reduced release of the encapsulated bacteria, and increased stability at various pH ranges (87, 88). Some examples of improved survival of probiotics bacteria encapsulated in alginate-chitosan microcapsules compared to alginate microparticles are (i) *Lactobacillus casei, Bifidobacterium longum, Bifidobacterium longum subsp. infantis* and *Bifidobacterium breve* in digestive juices (83), (ii) *Lactobacillus casei* and *Lactobacillus acidophilus* in yogurt and milk (89), (iii) *Lactobacillus acidophilus* in simulated gastric solution (90), (iv) *Bifidobacterium breve* in simulated gastric solution (91), (v) *Bifidobacterium longum* in gastrointestinal fluids and at elevated temperature conditions (92).

Examples of the increased survival in simulated gastric solution and bile salts of probiotics encapsulated in alginate-composite compared to alginate microparticles only are (i) *Bifidobacterium bifidum* loaded in alginate-coated poly-L-lysine microcapsules (93), (ii) alginate-pectin composite with encapsulated *Lactobacillus casei* (94), (iii) *Lactobacillus acidophilus and Bifidobacterium animals subsp. lactis* encapsulated in alginate-modified starch composite (95), (iv) *Lactobacillus delbrucekii* encapsulated into succinylated alginate (96), (v) *Lactobacillus plantarum* encapsulated in alginate coated with whey protein (97, 98), (vi) *Lactobacillus delbrueckii subsp. bulgaricus* and *Lacticaseibacillus paracasei* encapsulated in alginate-whey protein isolate microcapsules (99).

Common materials for protein-based probiotics encapsulating materials are casein, gluten, albumin, and whey proteins. Whey proteins are often the preferred source for ready-to-drink protein beverages because of their excellent nutritional qualities, bland flavor, ease of digestibility, and unique functionality in beverage systems (62, 100). For example, *Bifidobacterium breve* R070 and *Bifidobacterium longum* R023 encapsulated into whey protein microcapsules exhibited significantly higher viability than those of free cells after 28 days in yogurt. Increased tolerance of bifidobacteria encapsulated in water-insoluble whey protein-based microcapsules to high acid environments indicated this approach is useful for probiotic delivery to the gastrointestinal tract (101).

Casein used as a matrix for protecting *Lacticaseibacillus. paracasei and Bifidobacterium lactis* during gastric transit improved cell viability in pH 2.5 buffer by 20% relative to the free cells (102). *Lactobacillus acidophilus* and *Bifidobacterium lactis* encapsulated in spray-dried coacervates of casein with pectin resulted in an excellent increase in cell survival at very low pH values (103), *Lactobacillus rhamnosus GG* encapsulated in microparticles with whey protein isolate enabled very good cell survival in ex vivo porcine gastric contents (104). It was shown that during incubation in the stomach loss of probiotic content was minimal but in the contents of the intestine a release over 30 min occurred.

The addition of proteins to the alginate matrix increases cell viability and encapsulation efficiency. *Streptococcus thermophilus* and a probiotic strain *Lactobacillus delbrueckii* subsp. *bulgaricus*, *Lactobacillus acidophilus* and *Bifidobacterium bifidum* were encapsulated in whey protein-alginate and whey protein hydrolysate-alginate (5). The authors concluded that the presence of proteins in an encapsulation matrix contributes to the mechanical properties of beads, fermentative activity, better acid and bile tolerance, as well as better survival of probiotics in the simulated gastrointestinal condition.

Many health benefits of probiotics are associated with their survivability and stability in carrier food and gastrointestinal conditions. Among many beneficial health effects, probiotic bacteria also present significant antioxidant abilities. *In vitro* and *in vivo* studies indicate that probiotics may reduce oxidative damage, and free radical scavenging rate, and modify the activities of key antioxidant enzymes in human cells (105). Various strains of lactobacilli and bifidobacteria exhibit remarkable antioxidant activity in the host intestine and promote the production of antioxidant enzymes helping to remove reactive oxygen species (106, 107). In a review paper Wang et al. (106) summarized the mode of action of probiotic bacteria in antioxidation (probiotics may modulate the redox status of the host via their metal ion chelating ability, regulate signaling pathways, enzyme-producing reactive oxygen species, and intestinal microbiota).

Besides numerous investigations on encapsulated probiotics’ health benefits, the antioxidant properties of encapsulated probiotics have been investigated to a lesser extent. Krunić et al. showed that enriching the alginate matrix with whey protein and whey protein hydrolysates can preserve the antioxidant capacity and stability of whey-based beverages (5). Encapsulation is not only a solution to the problem of preserving the viability of probiotics, but can also increase the antioxidant properties of functional drinks. The most frequently used probiotics in fermented foods and beverages are lactic acid bacteria partially owing to their antioxidant properties (107). The authors pointed out that future research should be focused on biopolymer research for the encapsulation of probiotics with antioxidant, anti-rheumatic, anti-inflammatory, and ACE inhibitory properties.

A relatively novel trend in the functional beverage design is the use of microcapsules containing both probiotics and prebiotics (known as synbiotics). Prebiotics are food ingredients that selectively promote the growth and activity of the beneficial bacteria in the gut over detrimental bacteria. There has been little published data on this topic, but some of the examples are: (i) optimization of *Lactobacillus casei, Lactobacillus acidophilus, Bifidobacterium longum* and *Bifidobacterium bifidum* encapsulated in alginate with prebiotics revealed improved protection and survival (108); (ii) presence of prebiotic, oligosaccharides, stimulates the growth of *Lactobacillus acidophilus* 5 encapsulated in alginate-chitosan microcapsules incorporated in orange juice (89); (iii) *Lactobacillus casei* 359 microencapsulated in whey protein concentrate with prebiotic inulin revealed very high survivability in litchi juice. During the encapsulation process, inulin protects the cells from drying providing tolerance to a higher temperature (109). In a review paper, Rovinaru and Pasarin presented the synbiotic concept, challenges for synbiotic formulation in fruit drinks and future perspectives (110). They concluded that the encapsulation of synbiotics is a successful improvement of the viability and stability of probiotics in fruit juices.

*In vivo* evaluation of probiotics encapsulation efficacy performed mainly on rats and mice revealed a direct and easy delivery of probiotic cells in the intestinal region (111, 112). The viability of microencapsulated probiotic cells after oral administration may be followed by molecular biological approaches real-time PCR (113) and fluorescence *in situ* hybridization (114). However, to obtain relevant results, detailed *in vivo* studies and models of the digestive system more similar to humans are needed (115).

Various research works revealed that microcapsules loaded with probiotics (microcapsule formulations) increase the viability of the encapsulated ingredient during processing and in the final product, a functional beverage. Besides enhancing probiotics viability during processing and storage, microcapsule formulations also provide targeted delivery ensuring desirable health-promoting effects of probiotics on the host. The supply of probiotics to the site of action not only depends on the amount of the probiotic in a beverage but also on its bioavailability. Bioavailability is the amount of a probiotic that enters the circulation when introduced into the body to have an active effect. The bioavailability of a probiotic after ingestion depends on its absorption and in part on the changes that occur during passage through the gastrointestinal tract (116).

The selection of the matrix materials, probiotics to be encapsulated, methods of microencapsulation and application are interdependent (117). To obtain a well-designed microcapsule formulation for a specific application, it is important to optimize parameters during microcapsule preparation bearing in mind the properties of probiotic and matrix components, the method used for encapsulation as well as regulatory requirements, cost, industrial scalability, consumer needs, and economic issues. Although different techniques and wall materials have been reported for the effective encapsulation of probiotics, maintaining the viability of probiotics remains a major challenge and further research is needed in this regard (115). Proper selection of microcapsule formulation variables helps in designing microcapsules with the desirable release. Designing optimal formulations of microcapsules that ensure sufficient survival of probiotics in a functional product, prevent the release of probiotics and reactions with other compounds of the base drink, and enable the action of probiotics only at the point of use requires an interdisciplinary approach as well as a good understanding of the molecular interactions of all present ingredients. Any probiotic delivery system should be designed so that the probiotics are released within the colon for their health-promoting effect. Microencapsulation is a complex area that includes scientific research on colloidal, physical and surface chemistry and microbiology. A better understanding of the complex interactions of probiotics with complex beverage matrices and the physiological mechanism involved when beverages pass through the gastrointestinal tract is needed to ensure the optimal viability of probiotics under all circumstances.

## 7 Conclusion

Acid whey, a byproduct of dairy processing, presents both challenges and opportunities due to its high organic load and valuable nutrient composition. This review explored innovative strategies for acid whey valorization, including biotechnological processing, fermentation, and ingredient recovery. Potential applications span food, beverages, animal feed, bioenergy, and even cosmetics. Despite challenges such as its high acidity, lactose content, and mineral concentration, acid whey can be effectively utilized to create value-added products, including protein powders, fermented dairy products, alcoholic beverages, and nutraceuticals. Biotechnological approaches, such as enzymatic conversion of lactose into galactooligosaccharides, further enhance its potential for use in functional foods. Additionally, recent research highlights ways to increase probiotic viability in functional beverages through microencapsulation. Functional beverages, fortified with bioactive compounds, including probiotics, require technological advancements to maintain probiotic viability.

Encapsulation techniques, such as using an alginate matrix enriched with whey, have been shown to improve the antioxidant capacity and stability of whey-based beverages. This approach not only enhances probiotic survival during storage and gastrointestinal transit but also contributes to the sustainability of whey utilization by addressing environmental concerns related to whey disposal. Encapsulation materials, including biopolymers such as polysaccharides and proteins, play a crucial role in protecting probiotics and ensuring their controlled release in the intestine. Various encapsulation methods, such as spray drying, extrusion, and coacervation, offer promising solutions for developing stable probiotic-enriched functional beverages.

Overall, acid whey valorization presents a sustainable solution to reduce dairy industry waste while creating high-value functional products. Future research should focus on optimizing processing techniques, improving probiotic stability, and expanding applications to maximize the potential of acid whey as a functional ingredient. By addressing the current challenges and leveraging biotechnological advancements, acid whey can be transformed into a resource that benefits both industry and consumers.

A promising method for improving the viability and targeted distribution of probiotics in functional foods and beverages is microencapsulation. Achieving the intended functional qualities and making efficient use of bioactive compounds depend on the choice of suitable microorganisms, encapsulating materials, and microencapsulation techniques.

Microorganisms: Because of their probiotic and antioxidant qualities, strains of Lactobacillus and Bifidobacterium are commonly employed.

### 7.1 Encapsulation systems

Commonly used matrices include alginate, chitosan, whey protein, and casein. Whey proteins provide superior nutritional characteristics, while alginate is used extensively since it is inexpensive and biocompatible. Probiotic survival can be further increased by adding prebiotics (synbiotics) or combining ingredients like alginate and chitosan.

### 7.2 Procedures

Spray drying, extrusion, emulsion, coacervation, and lyophilization are examples of common procedures. The best methods for preserving probiotic viability are gentle ones.

The optimization of microcapsule preparation parameters, including the properties of probiotic and matrix components, regulatory requirements, cost, and consumer needs, is essential for designing effective microcapsules. Future research should focus on biopolymer research for encapsulating probiotics with various beneficial properties and on gaining a better understanding of probiotic interactions within complex beverage matrices to ensure optimal viability and health-promoting effects. *In vivo* studies and models that closely mimic the human digestive system are also needed for relevant results.
